# Multi-Agent-Based Simulation of a Complex Ecosystem of Mental Health Care

**DOI:** 10.1007/s10916-015-0374-4

**Published:** 2015-11-21

**Authors:** Alan Kalton, Erin Falconer, John Docherty, Dimitris Alevras, David Brann, Kyle Johnson

**Affiliations:** IBM Research, Nairobi, Kenya; Medical Affairs, Otsuka America Pharmaceutical, Inc., Princeton, NJ USA; Otsuka Pharmaceutical Development & Commercialization, Inc., Princeton, NJ USA; IBM Global Business Services, West Chester, PA USA; IBM Global Business Services, Costa Mesa, CA USA; IBM Global Business Services, Pittsburgh, PA USA

**Keywords:** Agent-based simulation, Mental health care, Model development, Psychiatric patient environment, Complex medical and social ecosystem, Care coordination, System management

## Abstract

This paper discusses the creation of an Agent-Based Simulation that modeled the introduction of care coordination capabilities into a complex system of care for patients with Serious and Persistent Mental Illness. The model describes the engagement between patients and the medical, social and criminal justice services they interact with in a complex ecosystem of care. We outline the challenges involved in developing the model, including process mapping and the collection and synthesis of data to support parametric estimates, and describe the controls built into the model to support analysis of potential changes to the system. We also describe the approach taken to calibrate the model to an observable level of system performance. Preliminary results from application of the simulation are provided to demonstrate how it can provide insights into potential improvements deriving from introduction of care coordination technology.

## Introduction

Understanding the impact of applying technology in a complex mental health ecosystem is a challenge. Mathematical modeling can help stakeholders develop, evaluate, and predict the impact without creating real-world disruption.

This simulation models changes introduced by care coordination technology (and associated process flows) predicting the impact on broader system characteristics such as emergency hospitalization, incarceration, homelessness and suicide. Care system changes include introduction of informed and coordinated engagement, diagnosis, and treatment delivered by medical, justice system and social service providers. The simulation model was built in parallel with the care coordination software, and the project recently received the attention of the United States House of Representatives [1].

Creating the simulation model involved multiple challenges including the acquisition and validation of data and process flows, calibration, and representation of comprehensive constituent elements of interest. The model was designed to encompass a population suffering from Serious and Persistent Mental Illnesses (SPMIs) and to simulate interactions and experiences across the medical, social, and criminal justice systems.

We selected an Agent-Based Simulation to model the granular interactions between individual patients and service providers. The agent-based framework was ideal for translating mental healthcare domain expertise to a computerized model and allowed stakeholders to understand how the model functions. Other researchers have used Agent-Based modeling to inform public policy and to recreate and understand complex healthcare systems. Axtell and Epstein’s innovative 1999 study [2] included an Agent-Based sensitivity analysis of potential Social Security policy changes on retirement age. Since then, the field of Computational Sociology has continued to expand and justify an Agent-Based approach to understanding a major U.S. city’s mental healthcare system [3].

In [4], the authors explain an Agent-Based approach to simulating a modern German hospital system. Many authors have since elaborated on the potential of Agent-Based modeling to inform improved healthcare decision making and outcomes [5]. Despite a growing body of Agent-Based models applied to physical health [6–8], little has been done to simulate mental health systems besides a very recent work by Silverman et al. [9]. Our work seeks to fill this gap by simulating an urban mental health environment. Of particular interest is simulating the impact of improved care coordination between mental health providers in a major metropolitan area: hospitals, prisons, community mental health centers, assisted living facilities and crisis stabilization units. This focus on care coordination is not directly addressed by Silverman et al. [9]. Leveraging over 125 h of consultation with police, social workers, hospital administrators, community care and housing facilities, and the criminal justice system, the authors designed and constructed the Agent-Based Simulation. The model framework is customizable to support multiple urban applications and includes over seventy-five input variables matched to real-world data and specific feedback from mental health experts and from academic literature [10–12]. Output statistics closely match measured real-world data evidencing the accurate recreation of the mental health environment in the subject urban area.

The following sections explain the design methodology, model calibration, and specific uses of the model to support analysis of potential care systems changes.

## Design methodology

Available systemic data for the mental health population is currently limited to reimbursement systems, legally stipulated criminal data, and state and locally mandated registration of specific activities. To compile the inputs to the logic and parameters of the model, information was collected through published statistics, interviews with medical personnel, police and criminal justice system personnel, and care services personnel [10–12].

The agent based framework was ideal for translating these detailed process flows and interviews with domain experts into a set of rules to be followed by a computational model. Agent based modeling also allowed the researchers to capture the dynamics of individual patients, both their internal dynamics of receiving and responding to treatment and their interactions with the larger ecosystem. As discussed below, tracking each patient as an agent in the model allows each agent to maintain their individual internal state, and use that internal state information as input to the wide variety of decisions that each agent makes on a weekly basis (up to 40 individual decisions each week). Other modeling approaches, such as system dynamics modeling, would have been a poor fit to the complexity and flexibility required to capture all of those individual decisions. Finally, it was important that the model be easily understandable to a non-technical audience. The unique ability of agent based models to visualize familiar processes and geographies shortened the learning curve when introducing the model to an unfamiliar audience.

## The model

Patients, care providers and other actors are represented as agents in the model. The agent’s environment captures key elements of larger mental health care ecosystems, including mental healthcare, living arrangements, employment, family support, and the criminal justice system. Factors in the environment, such as funding level, availability of staff, etc. dictate the frequency and effectiveness of interactions between patients and care providers. A rich set of input parameters help customize and tune the model to accurately represent current and/or target state conditions for a given locality.

During the course of simulation runs, mental health status, physical health status, and physical environments of patients are tracked, as well as system statistics like patient quality of life and recidivism. Costs are accumulated for individual services and for the overall system. Model runs are set for 5 years to capture long term system behavior. The model was built with AnyLogic Simulation Software.

Patients are created with randomly determined sets of characteristics based on input parameters. A patient’s score for mental health state is changed during the simulation run according to several key factors, including adherence to medication, interactions with care providers, and their environment. Each patient is assigned a minimum mental health state score, capturing variability in severity of patient conditions, recognizing that if left untreated, patients may decline to certain levels. Mental health of untreated patients degrades over time, impacted by factors such as stress, support, medicines, and interactions with care providers.

Each patient is assigned an initial physical health score that changes over the simulation run. Patients start with an initial physical health state, and are assigned a factor controlling how physical health changes over time. Physical health decline is influenced by the mental illness as well as environment. Mental and physical scores interact, modeling how treatment for severe mental illness can impact physical health and how physical conditions can impact mental health.

Both the mental and physical health scores operate on a scale of 1–100, with the mental health score approximating the Global Assessment of Functioning scale previously commonly used by mental health practitioners [13]. The model accounts for known factors impacting physical health and functioning.

Over the course of the model, a patient may transition to a different physical environment according to a defined set of probabilities that vary by mental health state. Each transition and environment affects mental and physical health states. A patient interacts with other actors with varying frequency, depending on his/her current mental and physical health state.

A patient is assigned a treatment plan, following it until changed by a care provider. Treatment plans are comprised of medicine regimes, services appointments, and physical environments. Appointments and other interactions between patients and care providers can raise or lower patient health.

The state of each patient agent is updated as the model progresses. Decisions take place in a rough sequence, which starts with a set of “critical outcomes” that determine if a patient is going to have a major change in state – e.g., entering a crisis state, starting to abuse substances, or finding employment. Over thirty consequential decisions are considered, reflecting logic developed during discussions with medical, social service and criminal justice advisors; each decision reflects combinations of internal patient state variables and model input variables.

**Fig. 1:**
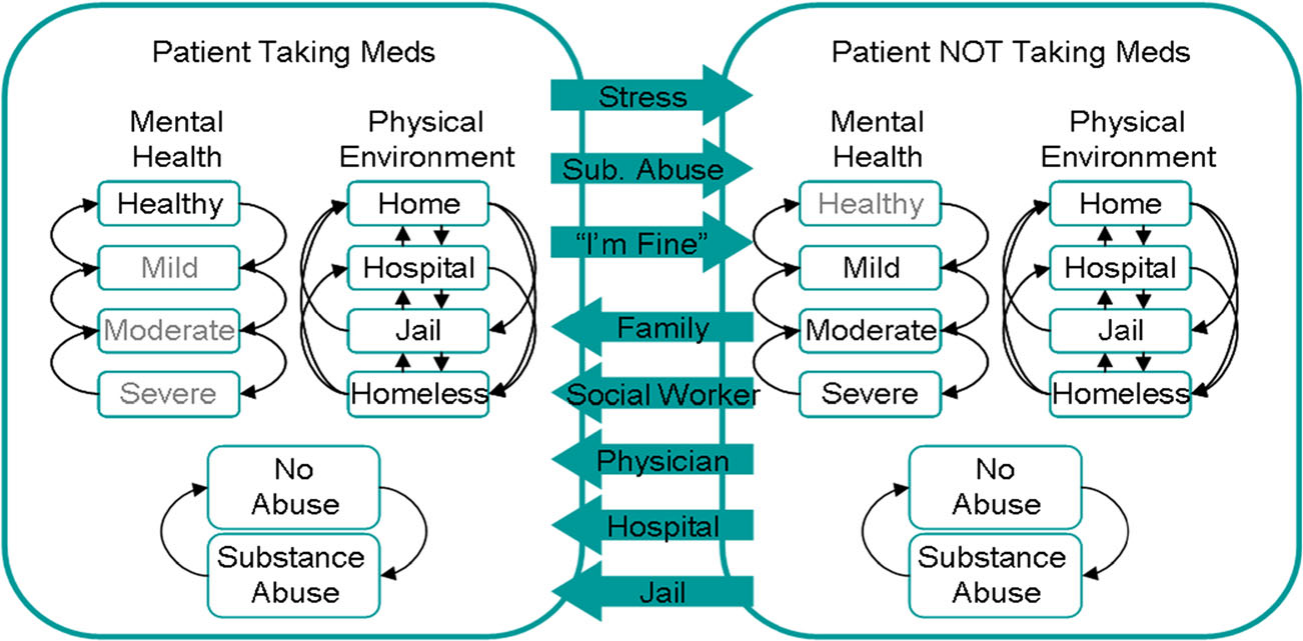
Illustrative patient state chart

The state chart in Fig. 1 represents a sample view of how one specific state of the patient is modeled - whether or not they are taking their medications. The outer boxes, or states, represent the primary options for that state, and the arrows between the two major states represent some of the factors that could drive a transition, such as stress, substance abuse, and family support. The actual model logic represents this state change based on a numerical score, where each of the factors contributes a positive or negative influence on that score.

Within each outer state are a number of related sub-states, modeled here as “mental health”, “physical environment”, and “substance abuse”. The patient transitions between these sub-states according to the factors described above.

The model supports a variety of care providers and services within the overall mental health care system. The number and density of these actors are controlled by model inputs, which will in turn dictate the probability of accessing a service when patients are instructed to by a treatment plan. The model primarily captures interactions of patients with the criminal justice system by tracking the Physical Environment of the patient. When patients enter a Correctional Facility, they are assigned a duration that controls how long they will remain in that physical environment. The patient is assigned a discharge location and treatment plan upon leaving the Correctional Facility.

The model captures a variety of financial factors, covering both the cost of care, and patient’s ability to afford care. Each patient is assigned an initial level of financial resources which can decline or improve during the model analysis period.

The various medicines, care providers, and services within the model have a cost-to-patient factor (0–5) associated with them, that impact a patient’s probability of accessing those services. They also have additional costs associated with them, such as costs borne by insurers, governments, and private organizations (e.g., churches). These are added to the costs borne by the patients in order to determine the total cost of care for the mental health care system.

Government and other funding is modeled as an overall “level of funding support” variable that influences the number and type of care providers, cost of access to medicines, cost of transportation, shelter etc. The funding level cost is modeled as support provided to individual components of the care delivery system, giving the model the ability to mimic funding cuts by state governments that affect Medicaid support or grants by charities that allow more case workers to operate in an area.

**Fig. 2:**
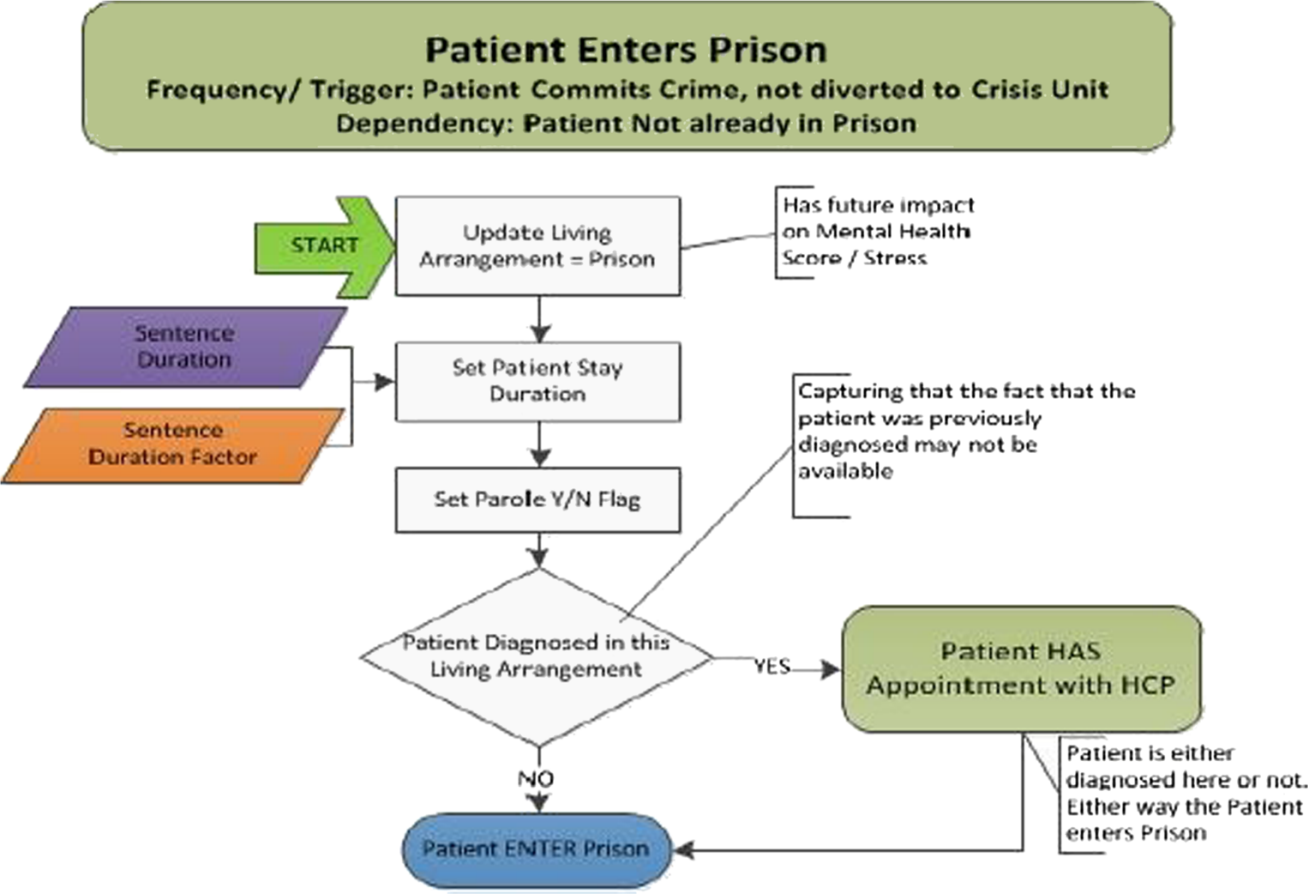
Simple decision flow. HCP = healthcare provider

Figure 2 illustrates a simple decision flow that a patient agent in the model might experience. This diagram provides an example of the framework used to document the model, both for the model developers, and also for validating the model behavior with the team of experts supporting the effort.

The simulation model is controlled by a rich set of input parameters. Patient data used for the background analysis was collected after obtaining appropriate consents and agreements, and was de-identified to protect patient privacy. These data are used to calculate probabilities of occurrence, or distributions and, where applicable, are provided by category (e.g., age, diagnosis etc.). Inputs include:

- System: length of the simulation and start/end dates.
- Patients: total population size, demographic data, adherence and behaviors, initial treatment plan, propensity to commit crime, probability of crisis onset, crisis outcome probabilities.
- Treatment Plans: medicine (generation, refill frequency, dosage, costs, formulary status, short and long term side effect characteristics), interactions/appointments, applicable physical environments.
- Care Providers and System Actors: number and capacity, probability of long acting injectable medication used at the facility, costs of services.
- Physical Environments: stability flag, probability of being located, capacity, length of stay.
- Law Enforcement and Criminal Justice: initial criminal history, CIT officer at crime scene, degree of crime, sentence durations, Jail Diversion Program entry.

**Fig. 3:**
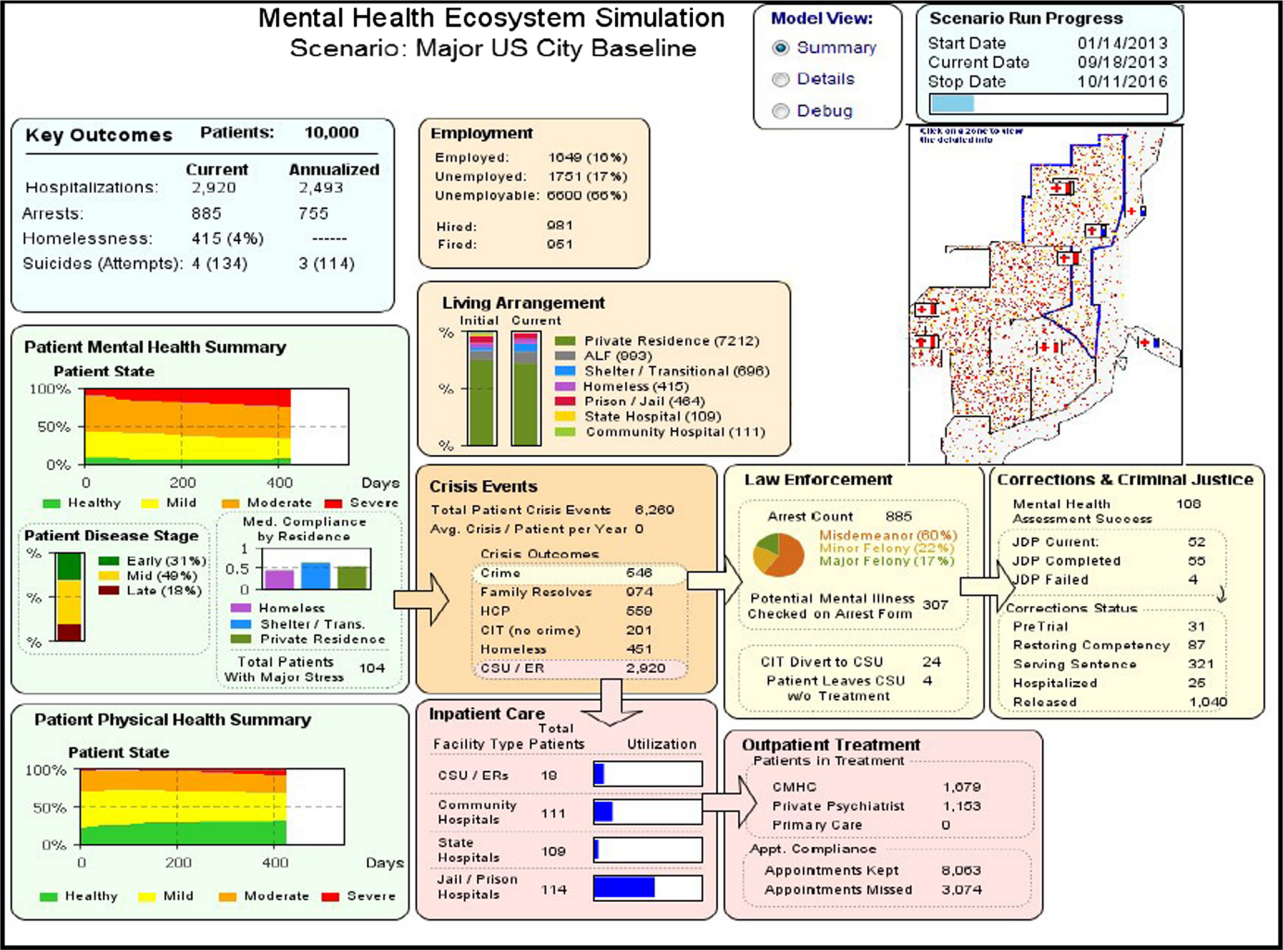
Simulation model animation

Key model output statistics are calculated and displayed during the simulation run and made available in an animated display, which was developed to aid in the development, verification, and validation (testing) of the simulation model. The animation, shown in Fig. 3, is a representation of the system and includes an illustrative representation of the size and state of the patient population and key system statistics. The simulation model exports a set of metrics including patients by mental health category, rates of recidivism, costs, and resource utilization. These metrics are calculated and exported to output files for analysis together with other statistics including adherence tracking.

Further information about the model’s internal structure can be found in this work’s supplementary material [14].

## Calibration

The calibration of the model followed a tiered approach, gradually fine-tuning the overall set of input parameters. The first tier of calibration was focused on tuning the model to match single high-level historical data points such as the total number of mental health crises in a year, selected due to high confidence in the accuracy of reference statistics, and because crisis is a key factor in model dynamics. This initial calibration phase aimed to achieve a +/−20 % accuracy, recognizing that this statistic represented an aggregate of a number of model outputs. Sanity checks on the other key model outputs where also completed to ensure that the model was consistent with expert assessments and reference data for the ecosystem.

The next level of calibration and validation looked at the key outcomes of a mental health crisis: levels of hospitalizations, arrests, homelessness, and suicides. These data points had available historical reference for calibration [10], and the metrics are representative of key outcomes within the model. Matching historical data contributed to improved overall confidence in the model as an accurate reflection of the ecosystem. Hospitalization and arrest metrics were also important indicators that the inpatient medical care and the judicial sub-system models were performing correctly. Once this calibration was successfully concluded, drivers for these key outcomes were investigated. For example, factors influencing hospitalizations include medication adherence, appointment attendance, and living arrangements. Each of these supporting drivers was calibrated to match data available in multiple academic reviews of SPMI population living arrangements [11, 12].

This calibration process was time-consuming, and adjusting inputs to modulate a single lower-level metric frequently often impacted beyond the single metric, leading to the need to balance across a number of inputs and outputs. A majority of the outputs were effectively calibrated, including distribution of living arrangements, crisis outcomes, law enforcement & criminal justice metrics, and inpatient treatment outcomes.

In addition to tracking the overall model outcomes, checks on the internal dynamics of the model were completed. This included examination of the variability of mental health scores over time. Model inputs and logic were adjusted where required to limit the dynamic nature of the variability for some classes of patients.

In many cases high-quality data was available for the specific metropolitan area (e.g., number of arrests, suicides, use of inpatient mental health beds). In other cases, data was available at a state or national level [10–12], in which case, the most directly-applicable data was selected. This data gathering approach applied to both the output metrics used for calibration as well as model input variables. Expert opinion gathered from discussions with medical, social service and criminal justice advisors was also used to supplement the knowledge required for the model development and calibration.

## Application and results

The simulation was applied to evaluate how care coordination technologies in specific areas would impact system performance. Example areas include the transfer of patients between facilities and improving patient attendance at appointments. An expert panel drawn from administrators and mental healthcare delivery personnel were engaged to describe the change in processes and estimate the possible range of improvements to specific parameters describing process efficiency and effectiveness. Process changes were then introduced within the simulation model, and iterations were run to capture the range of parametric changes required.

### Improvement in patient transfer between providers

One major issue in current healthcare delivery is losing track of patients during transfers between providers. Patients that disappear do not receive treatment required to manage their conditions, leading to crisis. The simulation introduces a *Handoff Success Rate* parameter that models this effect. Care coordination technology shares information about patients during a referral transition, allowing receiving facilities to gain awareness and reach out to incoming patients to improve transfer success.

By improving the *Handoff Success Rate*, the model assesses the impact of improved continuity in patient treatment across facilities, examining the impact in terms of improvements in patient care and reduction in system crisis events such as incarceration and hospitalization. The expert panel discussed how technology driven knowledge transfer enables better patient coordination, estimating the range of change to effectiveness, represented by a percentage point change of +5 to +15 % for the *Handoff Success Rate*.

The simulation was run adjusting the *Handoff Success Rate* parameter by 1 % intervals from −15 to +20 % to characterize the relationship between the parameter and output measures.

**Fig. 4:**
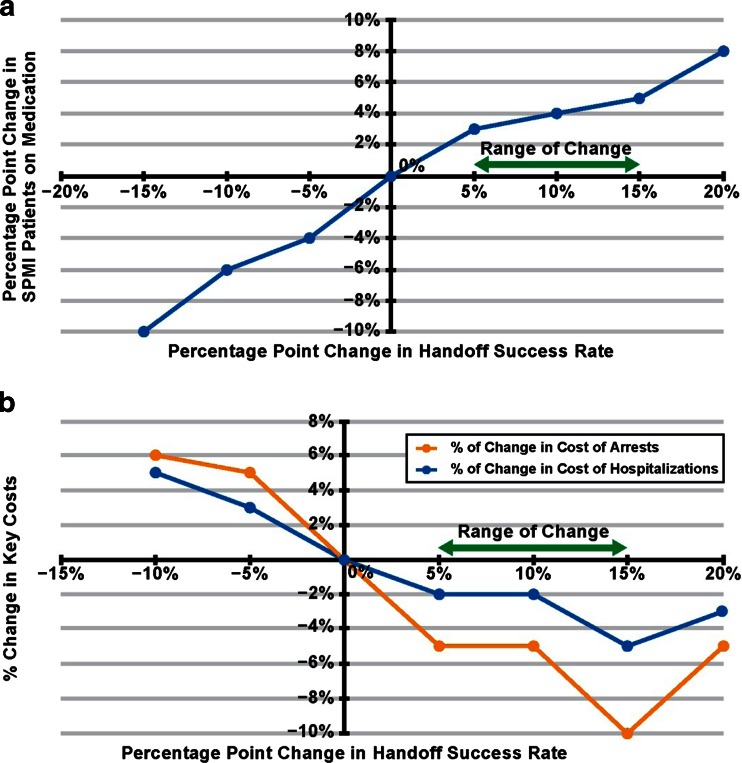
The effect of referral process improvements on percentage of persons with SPMI medicated (**a**) and key costs (**b**). SPMI = serious and persistent mental illness. Range of Change = expert panel-predicted range of impact of technology-driven care coordination

We selected three output measures – 1) medication compliance/patients on medication which charts a primary goal for successful transfer of ensuring continuity of medication, and the cost of failures which tabulated changes to costs of 2) incarceration and 3) hospitalization. As shown in Fig. 4a, medication compliance/patients on medication showed a 3.5 to 5.5 % improvement across the predicted range of change to effectiveness. Reductions in the costs of failure ranged from 5 to 10 % for incarceration while hospitalization costs were about half that level (Fig. 4b). This suggests a primary crisis point for “lost” patients as the criminal justice, rather than healthcare system.

### Improvement in patient appointment compliance

Appointment compliance is also a key issue, estimated to be less than 50 %. The simulation introduces a parameter *Patient Appointment Compliance Rate* to model this. Care coordination technology can improve appointment management by introducing reminders and alerts, through better planning processes, and through coordinating transportation. The expert panel discussed how these kinds of technology driven capabilities can affect appointment compliance, estimating a range of change, represented by a percentage point change of +3 to +12 % for the *Patient Appointment Compliance Rate*.

**Fig. 5:**
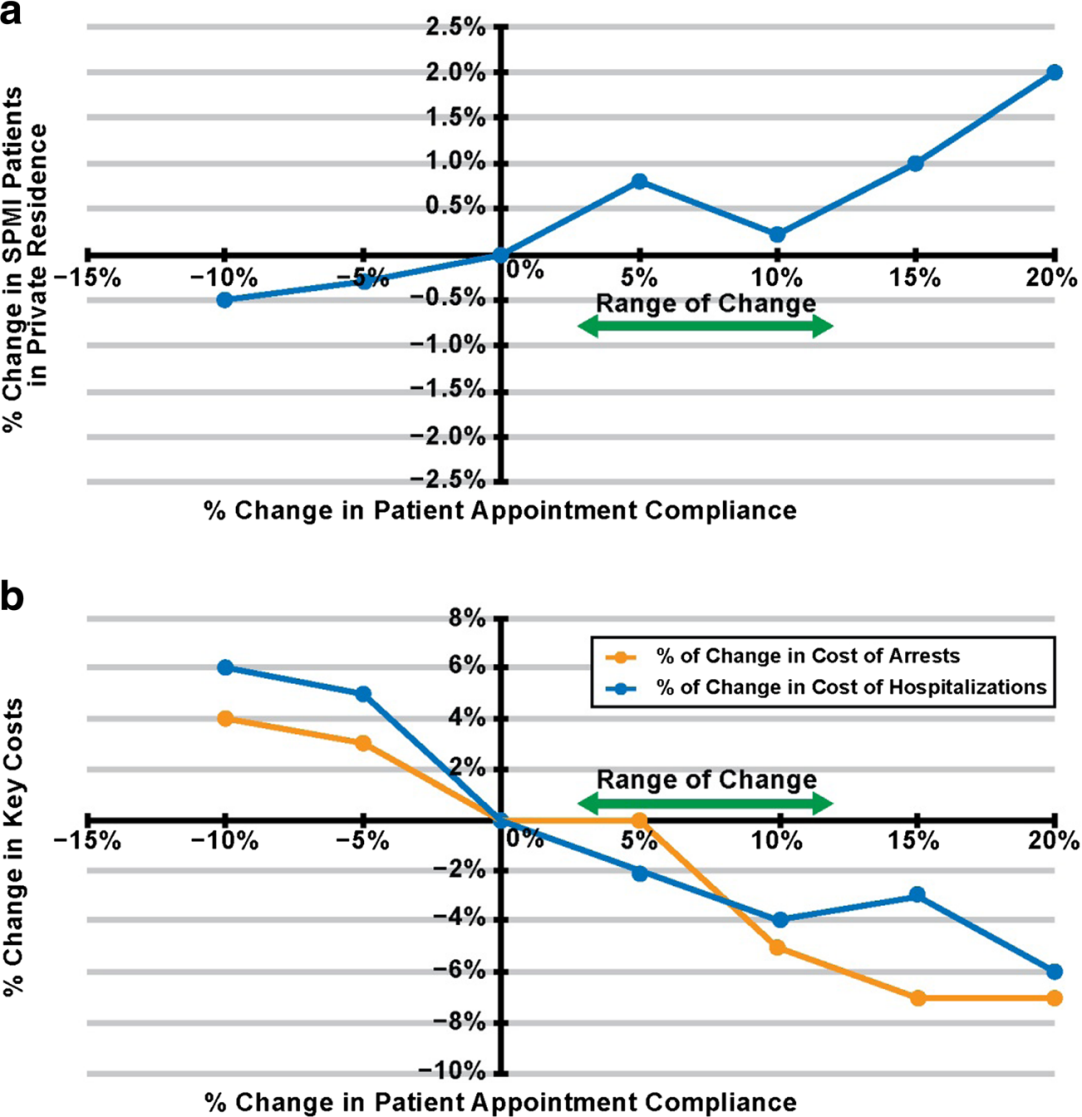
The effect of improved appointment compliance on the number of persons with SPMI living in private residence (**a**) and key costs (**b**). SPMI = serious and persistent mental illness. Range of Change = expert panel-predicted range of impact of technology-driven care coordination

The simulation was run adjusting the *Patient Appointment Compliance Rate* parameter by 1 % intervals from −10 to +20 % to again characterize the relationship between the parameter and output measures. We select three output measures – 1) number of patients living in private residence (a primary goal for patients in stable treatment programs), and the cost of crisis which tabulated changes to costs of 2) incarceration and 3) hospitalization. As shown in Fig. 5, the number of patients living in private residence increased by 0.25 to 0.75 % across the predicted range of change. Reductions in the cost of crisis ranged from 1 to 3 % for both incarceration and hospitalization.

## Conclusions

Fragmentation of care contributes to inefficiencies in and poor outcomes of mental healthcare systems. Inefficient allocation of resources and inadequate, discontinuous treatment processes lead to increased patient suffering and relapses, more emergency service utilization and increased incarcerations.

The use of an Agent-Based Simulation model helps represent complex ways in which patients engage with medical and social ecosystems. It has been used to predict impact of care coordination technologies, allowing system managers to analyze the overall dynamics and system performance across changes to social, medical and criminal justice components. The model also helps system managers test hypotheses about changes to care delivery and treatment planning, set productivity expectations, and explore reasons for observed deviation.

The results presented here represent preliminary examples of analyses that estimate impact of care coordination technology. As these technologies are introduced, further analysis is planned to compare realized impact with the predictions. Additional modeling considerations will be required to capture phased and differing technology adoption profiles for agents. Work is also ongoing to adapt the model to represent new metropolitan areas in the United States. The basic model construct is proving adaptable, helping to structure the approach to data capture; however modeling of different policies, resources and funding approaches needs to be further explored.
